# Effects of saponins from Chinese herbal medicines on signal transduction pathways in cancer: A review

**DOI:** 10.3389/fphar.2023.1159985

**Published:** 2023-03-29

**Authors:** Mingtao Zhu, Yanping Sun, Haodong Bai, Yimeng Wang, Bingyou Yang, Qiuhong Wang, Haixue Kuang

**Affiliations:** ^1^ Key Laboratory of Basic and Application Research of Beiyao (Heilongjiang University of Chinese Medicine), Ministry of Education, Harbin, China; ^2^ School of Traditional Chinese Medicine, Guangdong Pharmaceutical University, Guangzhou, China

**Keywords:** triterpenoid saponins, signal transduction pathway, anti-cancer, Chinese herbal medicine, steroid saponins

## Abstract

Cancer poses a serious threat to human health, and the search for safe and effective drugs for its treatment has aroused interest and become a long-term goal. Traditional Chinese herbal medicine (TCM), an ancient science with unique anti-cancer advantages, has achieved outstanding results in long-term clinical practice. Accumulating evidence shows that saponins are key bioactive components in TCM and have great research and development applications for their significant role in the treatment of cancer. Saponins are a class of glycosides comprising nonpolar triterpenes or sterols attached to hydrophilic oligosaccharide groups that exert antitumor effects by targeting the NF-κB, PI3Ks-Akt-mTOR, MAPK, Wnt-β-catenin, JAK-STAT3, APMK, p53, and EGFR signaling pathways. Presently, few advances have been made in physiological and pathological studies on the effect of saponins on signal transduction pathways involved in cancer treatment. This paper reviews the phytochemistry and extraction methods of saponins of TCM and their effects on signal transduction pathways in cancer. It aims to provide theoretical support for in-depth studies on the anticancer effects of saponins.

## Introduction

Cancer is one of the most lethal diseases caused by cells escaping homeostatic control and proliferating and differentiating abnormally. It is the second leading cause of human death worldwide (Yan et al., 2017). As of 2019, approximately 23.6 million new cancer cases and 10 million cancer deaths were reported worldwide, and the trend has been increasing annually (Kocarnik et al., 2022). Among them, lung cancer is the leading cause of morbidity and mortality among men, and global cancer data published by the International Agency for Research on Cancer (IARC) (2020) indicate that lung cancer ranks first in terms of morbidity and mortality among all malignancies (Sung et al., 2021). Breast cancer is one of the most common malignant tumors among women, with the highest incidence rate (Arnold et al., 2022). Other types of cancers, including those of the colon, esophagus, and pancreas, are also increasing yearly, thereby seriously endangering the physical and mental health of human beings. Currently, the main method of cancer treatment is chemotherapy (Nagasaka and Gadgeel., 2018), and the commonly used chemotherapeutic agents are cytotoxic and antimetabolic drugs, including adriamycin, cyclophosphamide, etc. (Khan et al., 2019); however, chemotherapeutic drugs can cause serious side effects of immunodeficiency, fatigue, diarrhea, and respiratory difficulties (Qiang et al., 2023). Therefore, the search for safe and efficient drugs or ingredients is a global concern.

**FIGURE 1:**
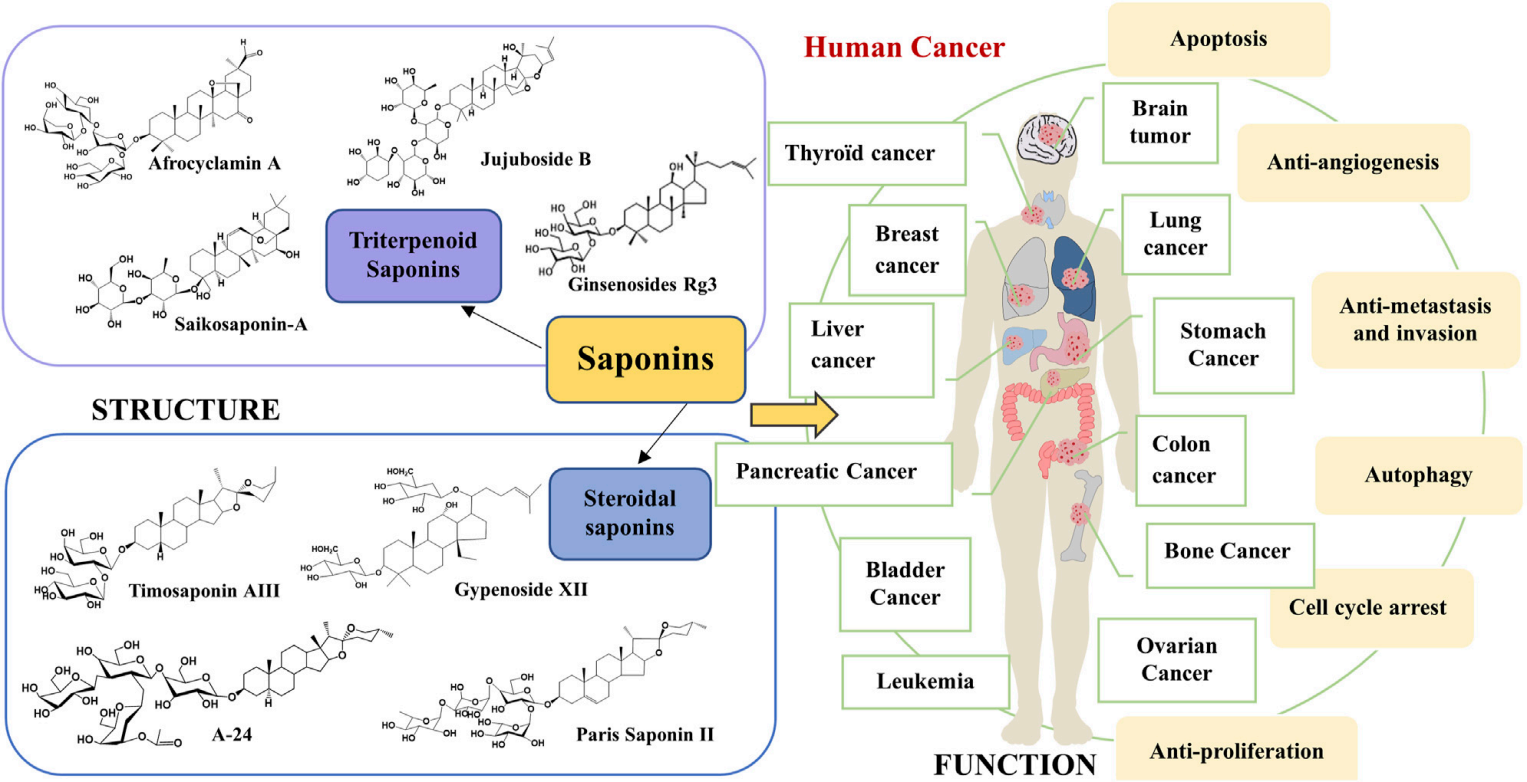
The therapeutic mechanism of saponins from Chinese herbal medicine on cancer.

Compared to traditional chemotherapeutic drugs for cancer treatment, active ingredients derived from Chinese herbal medicine (CHM) including saponins, polysaccharides, alkaloids, flavonoids, volatile oils, etc., have the advantages of multi-target synergistic effects and less toxic side effects, thus effectively inhibiting cancer cells from invading (Kumar and Jaitak., 2019) or metastasizing and differentiating (Sarwar et al., 2018; Zhou et al., 2018). Saponins are a class of glycosides with relatively complex structures and are widely found in CHM, such as *Panax ginseng* (Wei et al., 2020), *Caulophyllum robustum* Maxim (Lü et al., 2019), etc. They exert a variety of important biological activities and have a wide range of pharmacological effects; for example, antitumor (Xu et al., 2016), immunomodulatory (Bhardwaj et al., 2014), antioxidant (Wang et al., 2015), anti-inflammatory (Xiang et al., 2016), hypoglycemic (Uzayisenga et al., 2014), and therapeutic in cardiovascular diseases (Wu et al., 2019). CHM is one of the most active and fastest-progressing areas of research in traditional Chinese medicine. Saponins not only have the advantages of multi-target and multi-pathway in Chinese medicine but the active ingredients are also easy to identify and refined in modern medicine. Accumulating evidence shows that saponins prevent and treat cancer through multiple mechanisms and links, mainly including the induction of cell cycle arrest, promotion of apoptosis, induction of autophagy, anti-angiogenesis, inhibition of migration, and induction of tumor cell differentiation (Tian et al., 2020; Chen et al., 2018a; Liu et al., 2014). The potential mechanism of action of saponins against cancer is shown in Figure 1. Saponins can reduce the side effects on patients by eliminating tumor cells through apoptosis (Man et al., 2010). Ginsenosides (Yao and Guan., 2022), astragalosides (Georgieva et al., 2021), *Pulsatilla chinensis* saponins (Li et al., 2020), *Paridis Rhizoma* (Chonglou) saponins (Yu et al., 2022), Radix Bupleuri saponins (Cheng and Ying., 2021), and *Zizyphus jujuba* saponins (Jia et al., 2020) have significant and excellent anticancer effects. Therefore, saponins are promising drug candidates in the biomedical and pharmaceutical fields.

In recent years, owing to the in-depth study of cancer pathogenesis, tumorigenesis is found to be related to the transduction of many signaling pathways, including JAK-STAT3, NF-κB, MAPK, p53, PI3Ks-Akt-mTOR, Wnt and others, and these are considered important in regulating key functions of human tumor cells (Zhu et al., 2022; Song et al., 2022; Kobayashi et al., 2020). Modifications of various key regulatory pathways promote tumor cell metabolism, proliferation, and apoptosis, and multiple alterations in cell signaling mechanisms cause changes in cancer cells. The complex nature of cell signaling networks is a useful attempt to better understand the behavior and biological processes of tumor cells (Fu et al., 2022). The use of key signaling molecules in the pathway as targets for drug therapy has been the focus and is a fundamental direction for studying the mechanism of drug action. However, the progress of research on the effects of saponins on the corresponding cellular signal transduction pathways in the anticancer process is poorly reported, which is a matter of concern.

Taken together, saponins, as active ingredients in CHM, are effective against different kinds of tumors. This paper presents a review of the phytochemistry of the saponins of CHM, their effect on cell signal transduction pathways, and the progress of research on their extraction methods since 2014. The data presented in this review were collected from different websites, including PubMed, Citexs, Web of Science, Elsevier, and Science Direct. The keywords used in our search were “saponins”, “anticancer potential of saponins”, “biological activities of saponins”, “signal transduction pathway”, “cancer”, “triterpenoid saponins”, etc. This review aimed to sort out the key signaling pathways involved in the functional mechanism of saponins from CHM for cancer treatment, and we hope to provide scientific information for researchers and clinical workers, for the in-depth research and exploitation of saponins.

### Saponins

Phytochemical studies have shown that saponins can be divided into the following two major groups: triterpenoid and steroid (Fuchs et al., 2017). Among them, triterpene saponins are the most widespread, and consist of 30 carbon skeletons of triterpene glycosides in a pentacyclic structure (Augustin et al., 2011). Many common herbs including those in the Araliaceae, Leguminosae, Polygalaceae, and Campanulaceae families, etc. Contain triterpene saponins, which can be divided into tetracyclic triterpenes and pentacyclic triterpenes (Han and He., 2021). Steroid saponins are a class of steroidal glycosides of spirostane compounds combined with sugars. These do not contain carboxyl groups in the molecules and are often neutral. Steroidal saponins are raw materials for the synthesis of steroid hormones and related drugs and are widely used in the pharmaceutical industry (Passos et al., 2022). Steroidal saponins are mostly found in plants belonging to Dioscoreaceae, Agavaceae, and Scrophulariaceae, and Liliaceae, Gingeraceae, and Trilliaceae *Lindl* families (Dong et al., 2019). Based on their structures, they can be classified into four groups, namely, spirostanol, isosprirostanol, furostanol, and pseudospirostanol.

### Composition

Saponins mainly comprise sapogenin with sugars, glyoxylates, or other organic acids. Among them, the sapogenin skeletons of triterpene saponins mainly include cycloastragenol, dammarane, oleanane, ursane, and lupane, and the sapogenin skeletons of steroidal saponins include spirostane, furostane, cholestane, and cardenolide (Zeng et al., 2022). The composition of sugars mainly includes glucose, galactose, rhamnose, arabinose, and other pentoses (Singh and Chaudhuri., 2018).

### Structure

**FIGURE 2:**
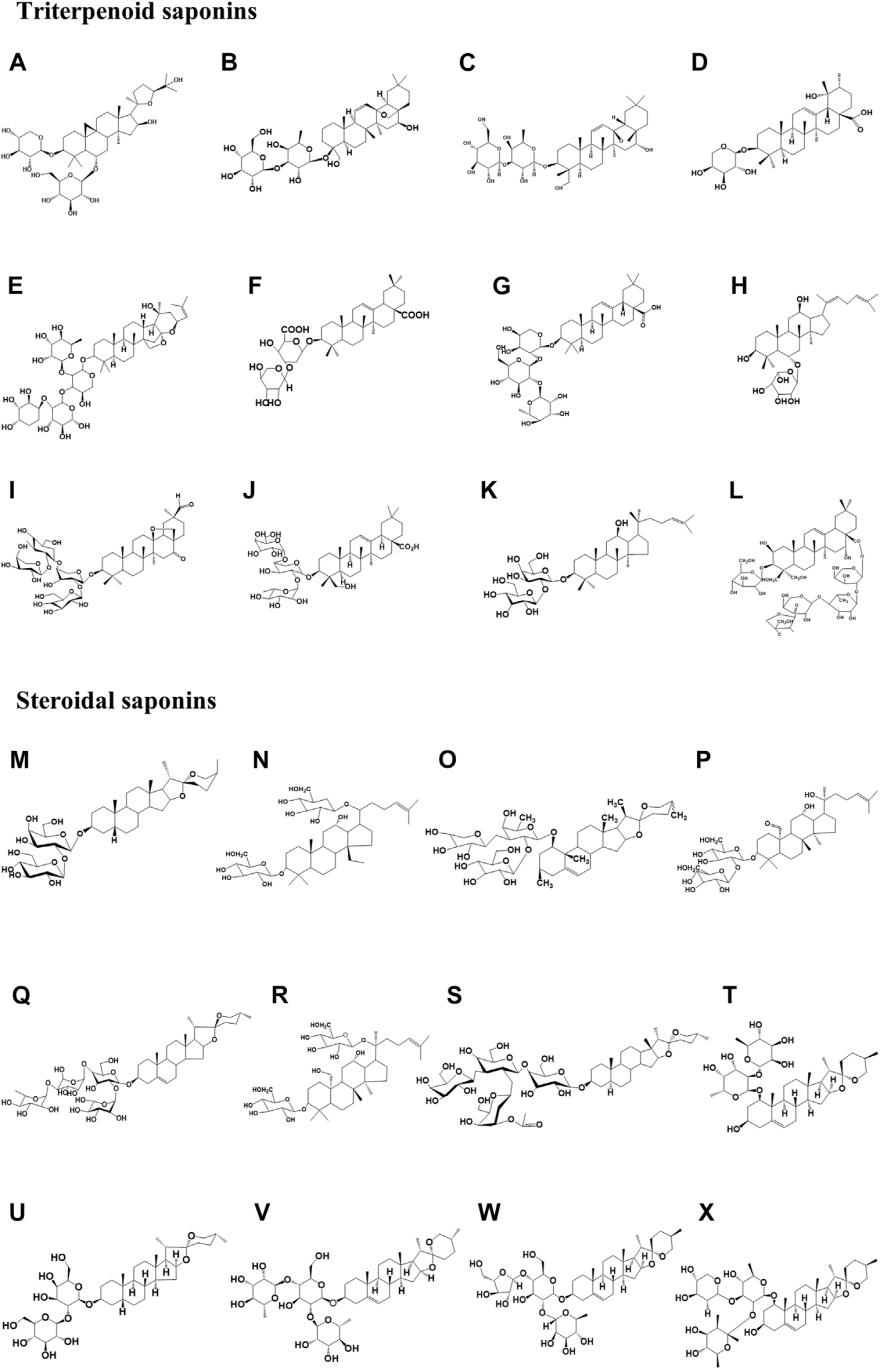
Chemical structure of triterpene saponins and steroidal saponins involved in the paper. Among them, **(A)** Astragaloside IV (Molecular formula: C_41_H_68_O_14_), **(B)** Saikosaponin-A (Molecular formula: C_41_H_66_O_13_), and **(C)** TSBE (Molecular formula: C_42_H_69_O_13_), **(D)** Ziyuglycoside II (Molecular formula: C_35_H_56_O_8_), **(E)** Jujuboside B (Molecular formula: C_52_H_84_O_20_), **(F)** Momordin Ic (Molecular formula: C_41_H_62_O_14_), **(G)** Raddeanin A (Molecular formula: C_46_H_74_O_16_), **(H)** Ginsenosides Rh4 (Molecular formula: C_35_H_58_O_8_), and **(I)** Afrocyclamin A (Molecular formula: C_54_H_86_O_20_), **(J)** Pulsatilla saponin D (Molecular formula: C_47_H_76_O_17_), **(K)** Ginsenosides Rg3 (Molecular formula: C_43_H_74_O_12_), **(L)** Platycodin D (Molecular formula: C_57_H_92_O_28_); **(M)** Timosaponin AIII (Molecular formula: C_39_H_64_O_13_), **(N)** Gypenoside XL (Molecular formula: C_42_H_70_O_14_), **(O)** DT-13 (Molecular formula: C_46_H_74_O_15_), **(P)** Gypenoside XII (Molecular formula: C_42_H_72_O_12_), and **(Q)** Paris Saponin II (Molecular formula: C_49_H_78_O_22_), **(R)** Gypenoside LXXIX (Molecular formula: C_42_H_72_O_14_), **(S)** A-24 (Molecular formula: C_55_H_90_O_21_), **(T)** Ophiopogonin B (Molecular formula: C_39_H_62_O_12_); **(U)** Timosaponin A3 (Molecular formula: C_39_H_64_O_13_), and **(V)** Dioscin (Molecular formula: C_45_H_72_O_16_), **(W)** Polyphyllin D (Molecular formula: C_44_H_70_O_16_), and **(X)** Ophiopogonin D (Molecular formula: C_46_H_74_O_14_).

The structures of natural glycoside components are mostly in the form of hydrophobic glycosides or triterpenoids or steroids connected to glycosyl groups by glycosidic bonds, and usually, the sugar chains are attached to the sapogenins either as a unilateral sugar chain (one sugar side chain at C-3) or a bilateral sugar chain, i.e., two sugar side chains at C-3 and C-28 (Baky et al., 2022). The cytotoxic activity of saponins is associated with the presence of a free carboxyl group at C-28 and a glycosyl group at C-3 (Cho et al., 2016). Among them, the free carboxyl group at C-28 is important for antitumor activity (Chwalek et al., 2006). Saponins have large molecular weights and complex structures, and often those with similar chemical structures are subject to small differences in the number of sugar chains and the location of the linkage sugars, resulting in different biological activities. Primary saponins can be converted into hypo saponins or sapogenins by enzymatic digestion, and by the intestinal flora (Luo et al., 2020; He et al., 2019a). Most sapogenins have higher biological activity than their proto-saponin forms. For instance, the antitumor activity of ginsenosides is in the following order: sapogenins > monoglycosides > disaccharides > triglycosides > tetrasaccharides (Navarro Del Hierro et al., 2018a). Intestinal microbes are considered the “second genome” of the human body (Sommer et al., 2017), and saponins are metabolized by intestinal microorganisms to produce new components. These are absorbed into the blood and undergo biotransformation. The types of biotransformation reactions mainly include glycosyl hydrolysis, redox, acetylation, rearrangement, etc. Among them, hydrolysis of sugar groups is the most common during biotransformation (Chen et al., 2018b). New ingredients and sapogenins are produced by physiological and biochemical processes and exert therapeutic effects. This is a more profound transformation mechanism in the body, which is conducive to deepening the understanding of the material basis of saponins. Simultaneously, the complex structure of saponins makes chemical synthesis more difficult, and their biotransformation by intestinal flora yields highly active and low-toxic metabolic components, which has become a development trend in this field. The chemical structures of various triterpene and steroidal saponins described in the paper are shown in Figure 2.

### PI3Ks/Akt/mTOR signal transduction pathway

The phosphatidylinositol 3-kinase (PI3K)/protein kinase B (Akt)/mammalian target of rapamycin (mTOR) signaling pathway, plays a key role in the formation and development of various diseases, including cancer (Ediriweera et al., 2019), neurodegenerative disorders (Fakhri et al., 2021), etc. PI3K is a class of lipid kinases further classified into three subclasses, namely, PI3KI, PI3KII, and PI3KIII. Activated PI3K further catalyzes phosphatidylinositol bisphosphate (PIP2) to phosphatidylinositol trisphosphate (PIP3), and PIP3 can activate Akt (Tan., 2020). Akt is an important signaling target downstream of PI3K and is divided into three main classes, namely, Akt1, Akt2, and Akt3 (Yu et al., 2018). PI3K-Akt and AKT/mTOR signaling pathways have key roles in cell survival (Tewari et al., 2022; Xie et al., 2017). mTOR is a serine/threonine kinase, a key protein essential for life processes. mTOR is also a downstream signal of the PI3K/Akt pathway and a key component of most signaling cascades (Rong et al., 2020). The PI3K/Akt/mTOR signaling pathway affects the tumor cell cycle, apoptosis, autophagy, and angiogenesis by altering the activity of its downstream effector molecules, and therefore, it may be an effective tool for targeted cancer therapy.

TBSE, the main active ingredient of Bupleurum, induces apoptosis in human colon cancer SW480 and SW60 cells through the PI3K/Akt/mTOR signaling pathway. It reduces the expression of the anti-apoptotic member, Bcl2, causes downregulation of expressions of PI3K, Akt, mTOR, and p-PI3K, p-Akt, p-mTOR, and increases the expression of Bax, cleaved caspase-3, cleaved caspase-9 (Zhang et al., 2022). Afrocyclamin A increases the expression of p53, p21, and Bax, in addition to upregulating the expression of cleaved PARP, cleaved caspase-3, and Cyt-c but decreases the levels of Bcl-2, cyclin E, cyclin D, cyclin B, and inhibits the expressions of MMP-2 and MMP-9 (Sachan et al., 2018). PNS (Liu et al., 2022a) inhibits the proliferation of Y79 cells and induces apoptosis through the PI3K/Akt pathway. It significantly increases the levels of cleaved caspase-3, cleaved caspase-8, cleaved caspase-9, and downregulates Bcl-2, PI3K, p-Akt (THR308), p-Akt (SER473), and p-mTOR levels. Wang found that Hederacolchiside A1 could promote the expression of cleaved caspase-3 and cytochrome, downregulate bcl-2 levels, and inhibit the phosphorylation of PI3K, mTOR, Akt, and P70S6K (Wang et al., 2018a). Total secondary saponin elevated the expressions of Bax/Bcl-2, Cyt-c, caspase-3, and caspase-9 by inhibiting the proliferation of MCF-7 cells and inducing apoptosis through PI3K/Akt/mTOR signal transduction (Zhang et al., 2020). Moreover, other triterpenoid saponins, such as Jujuboside B (Li L et al., 2021) and Saikosaponin-A (Du et al., 2021) exert anti-cancer effects through the PI3K/Akt pathway.

**FIGURE 3:**
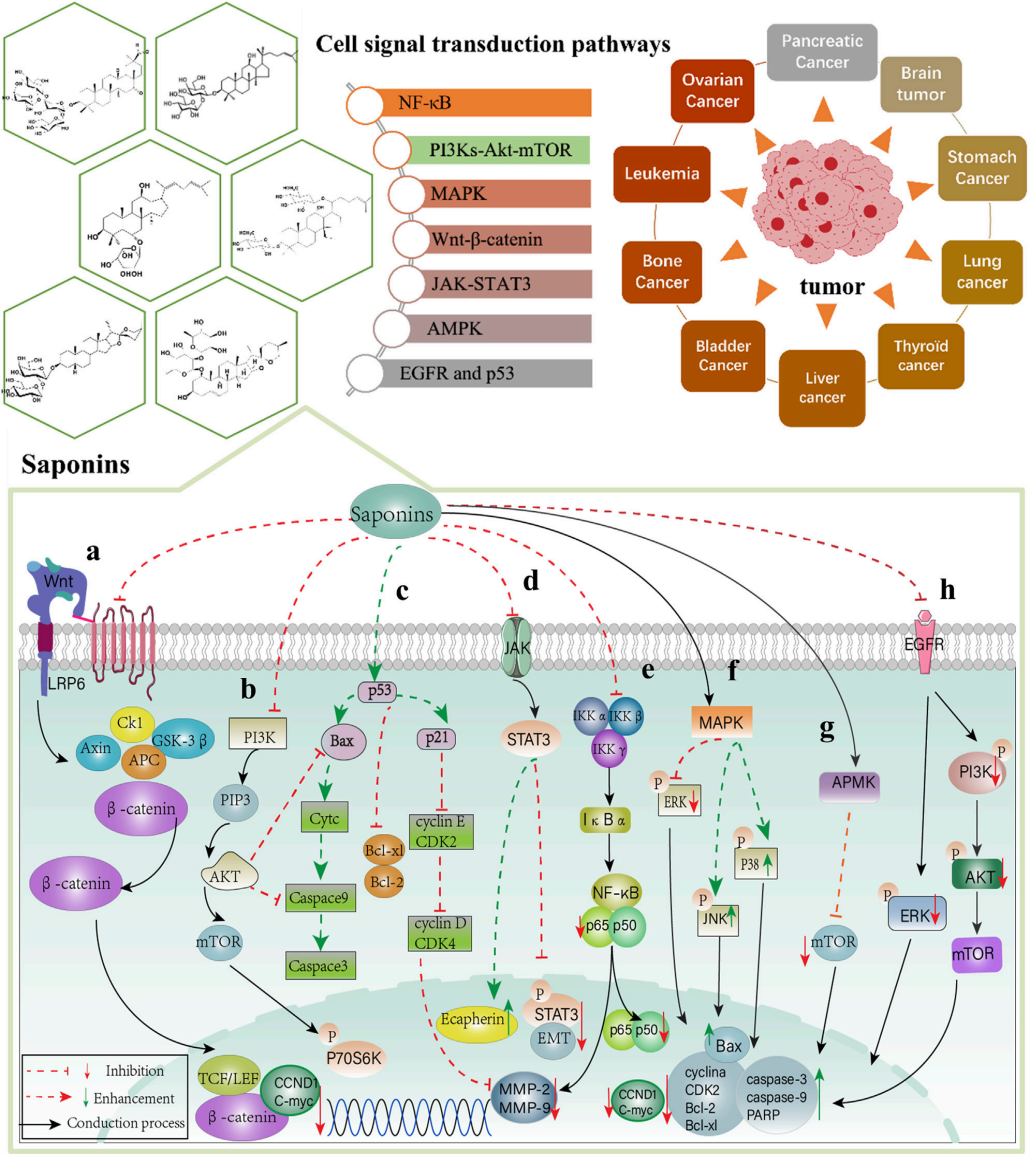
Signaling pathway of Chinese materia medica saponins in anti-cancer effect. **(A)** Wnt-β-catenin; **(B)** PI3Ks-Akt-mTOR; **(C)** p53; **(D)** JAK-STAT3; **(E)** NF-κB; **(F)** MAPK; **(G)** APMK and **(H)** EGFR signal transduction pathway.

Steroidal saponins, the material basis of higher plants like *Ophiopogon japonicus* (T.f) Ker-Gawl (OJ), *Anemarrhena asphodeloides*, and Rhizoma Paridis, inhibit cell proliferation (Wang et al., 2021a; Wang et al., 2020), induce cell cycle arrest (Long et al., 2015), and induce apoptosis (Liu et al., 2021; Song et al., 2019) through the PI3K/Akt/mTOR pathway. SSOJ significantly inhibits the expression of Ki67, p-PI3K/PI3K, p-Akt/Akt, and mTOR, and upregulates the levels of p53 and autophagy mediators (LC3-II/I ratio, ATG-3, ATG-7, and Beclin-1) (Chen et al., 2017). Paris Saponin II enhances the activities of Cyt-C, caspase 9, cleaved-caspase3, and Bax, while increasing the expressions of LC3-II and Beclin-1 by PSII but decreasing those of P62 and bcl-2, which are involved in PI3K/Akt/mTOR signaling (Zhang et al., 2016). Xu et al. showed that A-24 not only increased the expressions of cleaved-caspase-3, cleaved-caspase-8, cleaved-caspase-9, and Bax in SGC-7901 and AGS cells but also increased the levels of LC3-II and Beclin-1, and resulted in the downregulation of Bcl-2 expression, which played a role in inducing apoptosis and autophagy (Xu et al., 2020a). According to the results of *in vitro* research, Dioscin decreased the concentration of CHK2, cyclin B1, and CDK1 while increasing the expressions of cyclinD1, Bak, Cyt-c, caspase-3, caspase-9 in the HepG2 cells but decreasing those of bcl-2, bcl-xl and P70S6K (Zhang Y. S. et al., 2018). In summary, saponins play essential roles in anti-tumor activity by regulating the PI3K-Akt-mTOR signal transduction pathway (Figure 3B).

### Wnt/β-catenin signaling transduction pathway

The wingless-related integration site (Wnt) signaling pathway is an important intracellular signaling cascade with a regulatory role in cell proliferation, apoptosis, and differentiation in tissues and organs, and many diseases occur due to mutations or dysregulation in the Wnt signaling pathway. The widespread potential of the Wnt pathway in cancer has been reported (Duchartre et al., 2016). The Wnt signaling pathway is divided into classical (β-catenin dependent) and non-classical (β-catenin non-dependent) forms, which are key pathways that control developmental processes and histomorphogenesis (Koni et al., 2020). The dysregulation of the classical Wnt/β-catenin signaling pathway is involved in the pathological processes of many types of cancer (Zhang et al., 2018a; Cao et al., 2018; Spaan et al., 2018). β-Catenin is a switch in the Wnt/β-catenin signaling pathway, and phosphorylation of β-catenin is influenced by glycogen synthase kinase 3β (GSK3β) and casein kinase 1α (CK1α) (Nusse and Clevers., 2017). When pathway activation or degradation complexes are abnormal, β-catenin fails to phosphorylate or degrade and accumulates in the nucleus. Further, excessive concentrations of β-catenin bind to T-cell transcription factor/lymphocyte enhancer factor (TcF/LEF) and form complexes that activate the downstream target genes (cyclin D1, CDKN1A) and promote tumorigenesis and development (Zhang and Wang, 2020).

Triterpenoid saponins, like Raddeanin A (RA), inhibit proliferation and induce apoptosis in the CRC model both *in vivo* and *in vitro*. RA reduces the expression of β-catenin in the nucleus and cytoplasm along with the levels of Bcl-2, c-myc, p-GSK-3β, and cyclin D1 but promotes the expression of Bax (Wang et al., 2018b). Compound 1C is a modified version of AD-2, which is a ginsenoside isolated from *P. ginseng*. In LNCaP prostate cancer cells, 1C upregulates the expression of p53 but downregulated those of β-catenin, TCF-4 protein, CCND1, and C-myc (Wang et al., 2018c). *In vivo* and *in vitro* studies indicate that ginsenosides from Korean Red ginseng decrease the expressions of LEF1, CMYC, and CCND1 by inhibiting the Wnt/β-catenin signaling pathway (Ham et al., 2019). Saponins can regulate Wnt/β-catenin signal transduction and exert anti-cancer effects (Figure 3A).

### NF-κB signal transduction pathway

The nuclear factor kappa-B (NF-κB) signaling pathway is intricately and closely linked to other cellular signaling pathways, and pathway activation is an important factor in promoting tumorigenesis and progression. Extracellular stimuli (bacteria, viruses, oncogenic molecules, etc.) can activate NF-κB. The NF-κB signaling pathway is involved in several functions related to proliferation, metastasis, and angiogenesis, which are required for cancer (Dimitrakopoulos et al., 2020). Studies have confirmed that NF-κB is a transcription factor with oncologic therapeutic potential and has been recognized for its important role in colorectal cancer (Plewka et al., 2018), gynecologic cancers (Harrington and Annunziata., 2019; Diéguez-Martinez et al., 2022), pancreatic cancer (Geismann et al., 2019), and other types of cancer. NF-κB is also an important linker between chronic inflammation and cancer and regulates the expression of a wide range of genes associated with immune and inflammatory responses. It importantly contributes to the pathogenesis of inflammation-driven diseases (Chauhan et al., 2022). NF-κB includes classical as well as non-classical pathways, comprising bridging molecules for receptor and receptor proximal signaling, the IκB kinase complex, IκB proteins (IκBα, IκBβ, IκBε, IκBγ, IκBζ, Bcl-3, p100, and p105) and NF-κB dimers (Colomer et al., 2017). NF-κB signaling plays a role in reducing cancer cell proliferation and metastases and promoting apoptosis in the pathological process of tumors by downregulating downstream genes (Soleimani et al., 2020).

The involvement of saponins in antitumor signaling contributes to a better understanding of their role in the regulation of NF-kB (Figure 3E). CBS is a triterpenoid saponin extracted from the Chinese medicinal material, Conyza blinii H. lev. In xenografted animal models of tumor and HeLa cells, CBS downregulated the expressions of nuclear-translocated p65 and molecules downstream of NF-κB (XIAP, Bcl-xL, MMP-2, MMP-9, COX-2, and cyclin D1) by inhibiting the NF-κB signaling pathway (Ma et al., 2017). RA, a triterpene saponin of Anemone raddeana Regel, possesses potent anti-tumor properties. Both *in vivo* and *in vitro* research shows that RA reduces the levels of p-IκBα, p65, MMP-2, and MMP-9 by inhibiting ROS/JNK and NF-κB signaling pathways (Ma et al., 2018). Saponins of Patrinia villosa decrease the levels of E-cadherin, N-cadherin, and NF-KBp65 in the CRC EMT model by inhibiting the NF-κB signaling pathway (Xia et al., 2018).

Paris polyphylla is usually used as a heat-clearing and detoxicating agent in traditional Chinese medicine (Tu et al., 2016; Guo et al., 2019) and Polyphyllin VII is one of the primary natural steroidal saponins in it. *In vitro* research indicates that Polyphyllin VII promotes the expressions of caspase-3, poly-(ADP-ribose) polymerase cleavage but suppresses those of p65, PI3K, (P)-PI3K, AKT, P-AKT, NF-κB, and P-NF-κB and inhibitor of caspase-activated DNase by attenuating the PI3K/Akt and NF-κB signaling pathways (He et al., 2020). Similarly, an *in vitro* experiment suggested that Paris Saponin II lowered the expressions of p65, c-myc, and cyclin D1 in HT 29 and HCT 116 cells by inhibiting the NF-κB signaling pathway, as evidenced by the suppression of IKKα phosphorylation and p65 nuclear translocation (Chen et al., 2019).

### EGFR signal transduction pathway

The epidermal growth factor receptor (EGFR) is a transmembrane receptor glycoprotein of the tyrosine kinase family. Many cell fate-specific activities are regulated by the EGFR cell signaling pathway, including cell growth, differentiation, metabolism, and proliferation (Kyriakopoulou et al., 2018). Mutations in components of the EGFR pathway are usually closely associated with several human malignancies (Isomoto et al., 2020), and EGFR signaling is upregulated in 20% of tumors (Kang et al., 2022), inducing proliferation and inhibiting apoptosis (Guan et al., 2017). The development of molecular agents targeting the EGFR pathway offers attractive avenues for anti-tumor effects, and the presently available EGFR tyrosine kinase inhibitors in widespread clinical use are gefitinib and erlotinib (Ayati et al., 2021). Both *in vivo* and *in vitro* experiments indicate that Ginsenoside Rg3 enhances the anti-cancer cell proliferation effect of erlotinib and erlotinib-induced apoptosis. Erlotinib/ginsenoside Rg3 treatment increases the protein levels of caspase-3, caspase-9, and PARP while decreasing those of p-EGFR, p-PI3K, and p-Akt in pancreatic cancer cell lines and BALB/c nu/nu male mice by inhibiting the EGFR/PI3K/Akt signaling pathway (Jiang et al., 2017a). Ziyuglycoside II (ZYG II) is an active ingredient in the treatment of digestive system cancers (HCC, CCA, EC, and PC), and functions by inducing cell cycle arrest and activation of mitochondria-dependent apoptosis. ZYG II inhibits EGFR and ERK1/2 protein phosphorylation while enhancing the expression of cleaved caspase-3 and cleaved PARP (Zhong et al., 2021). Moreover, *in vitro* research suggests that Saikosaponin-d reduces the levels of EGFR, p-EGFR, MEK, p-MEK, p38, and p-P38 but promotes that of p53 in human RCC cells (769-P and 786-O) through the inhibition of the EGFR/p38 signaling pathway (Cai et al., 2017) (Figure 3H).

### JAK/STAT3 signal transduction pathway

The Janus kinase/signal transducer and activator of the transcription 3 (JAK/STAT3) signaling pathway has an important role in tumor behavior and function. STAT3 is a common signal transducer and activator of transcription that is involved in multiple signaling cascades. STAT3 deletion is a driver of tumor growth, and metastasis (Bharadwaj et al., 2020). STAT3 is one of the key oncogenes and therapeutic targets (Wang et al., 2021b; Chai et al., 2016). Recent evidence suggests that STAT3 is a regulatory node of cancer-related inflammation and a regulator of immune checkpoint proteins (Huynh et al., 2017). Autophagy and STAT3 pathways are two important directions in tumorigenesis and progression, and both have become research hotspots for tumor mechanisms in recent years (Hu et al., 2020; Jacquet et al., 2021). Autophagy, also known as type II programmed cell death, is a biological process important for maintaining tissue stability and metabolism. Abnormalities in autophagy are accompanied by alterations in STAT3 expression, and Xu et al. showed that STAT interacts with autophagy depending on several factors including phosphorylation sites, mode of action, and subcellular localization (Xu et al., 2022). STAT3 is a member of the STAT family and an important part of the JAK/STAT3 signaling pathway. Saponins were found to exert inhibitory effects on cell proliferation and induction of apoptosis through the modulation of the STAT3 pathway (Li et al., 2018; Li X et al., 2021; Zhou et al., 2019).

JAK/STAT3 is mainly divided into receptor tyrosine kinases (RTKs), JAKs (JAK1, JAK2, JAK3, and TYK2), and signal transducer and activator of transcription (STAT), which are activated by a variety of cytokines. Among them, the JAK2/STAT3 pathway is a component of JAK/STAT signaling that is upregulated in a variety of tumor cells and is particularly involved in the development of some solid tumors through the regulation of tumor cell proliferation and apoptosis (Jiang et al., 2016; Iriki et al., 2017; Wu et al., 2017). α-Hederin, a monodesmosidic triterpenoid saponin, is isolated from the leaves of *Hedera helix* and exerts potential anti-tumor effects in colon cancer. *In vitro* studies show that α-Hederin upregulates the expression of Ecapherin but downregulates those of IL-6-induced EMT markers (N-cadherin, vimentin, fibronectin, twist, and snail) by regulating the JAK2/STAT3 pathway to intervene metastasis in colon cancer (Sun et al., 2018).

Steroidal saponins, including DT-13, dioscin, and Ophiopogonin B show anti-metastasis and anti-proliferation effects, as do triterpenoid saponins. According to *in vivo* and *in vitro* studies, DT-13, isolated from the Dwarf lilyturf tuber, inhibits the phosphorylation levels of STAT3 and AKT in breast cancer cells (DA-MB-231 and MDA-MB-468) and reduces the expressions of the Procollagen-lysine, 2-oxoglutarate 5-dioxygenase 2 (PLOD2), and two receptors (Gp130 and OBR) by inhibiting the JAK/STAT3 and PI3K/AKT signaling pathways (He et al., 2019b). Moreover, both *in vivo* and *in vitro* experiments suggest that dioscin can upregulate the expressions of IL-4 and IL-10 by regulating the JAK2/STAT3 signaling pathway, thus inhibiting metastasis of B16 cells (Kou et al., 2017). Moreover, a previous study revealed that Ophiopogonin B could downregulate the protein expression of P-STAT3 by regulating the STAT3 signaling pathway, thus inducing apoptosis and affecting the cell cycle in SKOV3 and A2780 cells (Yuan et al., 2022). Steroidal saponins, the main active ingredient in Rhizoma Paris, are mainly composed of four kinds of saponins (Paris saponin I, II, VI, and VII). It is widely applied to treat tumors in China (He et al., 2019c). Paris saponin I can lower the levels of MMP-2, p-JAK2, and STAT3 in HUVEC cells (Wang et al., 2018a) (Figure 3D).

### MAPK signal transduction pathway

The mitogen-activated protein kinase (MAPK) signaling pathway can affect several different biological processes in cancer, including proliferation, differentiation, apoptosis, inflammation, and immunity (Wang et al., 2021c; An et al., 2020; Zhu et al., 2020; Lu et al., 2019), and plays a non-negligible role in the development of tumors. MAPK cell signaling mainly regulates the response of tumor cells to many internal and external stimuli (Anjum et al., 2022) In several malignancies, saponins regulate the MAPK signaling pathway through the induction of apoptosis and autophagic responses, thus exerting anticancer effects. Momordin Ic, a principal triterpene saponin constituent, isolated from Fructus Kochiae directly induces autophagy of hepatocellular carcinoma cells *in vitro*. Momordin Ic increases Beclin1 and LC-3 protein expressions in HepG2 cells by activating ROS-mediated JNK and p38 signaling pathways and regulating the ERK signaling pathway (Mi et al., 2016). Si et al. conducted *in vitro* experiments using human laryngeal squamous cell carcinoma cells, Hep-2 and TU212, and demonstrated that dioscin could exert potential anti-migration and anti-invasion, cell cycle arrest, and pro-apoptosis effects by significantly downregulating the protein and mRNA levels of cyclina, CDK2, Bcl-2, MMP2, and MMP9 while upregulating those of p53, Bax, Cyto-c, and caspase-3, caspase-9, p-JNK, and p-p38 by targeting the MAPK signaling pathway (Si et al., 2016). Treatment with water-soluble Astragaloside IV (AS-IV, 20 mg/kg) prepared from the roots of *Astragalus membranaceus* can inhibit proliferation and invasion *in vitro*. Furthermore, AS-IV reduces the expressions of MMP-2/9 and VAV3 in MDA-MB-231 cells by regulating the MAPK pathway, thereby downregulating tumor cell viability and growth, which is a potential strategy for treating metastatic breast cancer (Jiang et al., 2017b). Similar observations were reported in U251 cells and tumor-bearing athymic BALB/c mice. AS-IV upregulates the levels of Ki67, MMP-2, and MMP-9 by regulating the MAPK/ERK signaling pathway, which further results in the suppression of tumor cell growth, migration, and invasion abilities (Li et al., 2017).

According to their biological functions, the MAPK family mainly includes extracellular signal-regulated kinase (ERK), p38 mitogen-activated protein kinase, and c-Jun amino-terminal kinase 1/2/3 (JNK1/2/3), and these are involved in carcinogenesis (Lei et al., 2020; Kumar et al., 2020). ERK promotes cell proliferation and is involved in apoptosis and differentiation (Xu et al., 2019). JNK can be phosphorylated and can activate several proteins (c-Myc, p53, Bcl-2 family in the mitochondria of cell death regulators, etc.), and these nuclear and non-nuclear proteins regulate many cellular responses including cellular proliferation, differentiation, and apoptosis (Bubici and Papa., 2014). p38/MAPK is a stress protein kinase with core components *α, β, γ*, and *δ*. Stress stimuli, pathogens, or pro-inflammatory factors can activate the phosphorylation of p38/MAPK, which is involved in tumor development and has key regulatory roles in eliciting apoptosis, immune responses, and inflammatory responses (Martínez-Limón et al., 2020). The three intracellular MAPK signaling pathways interact with each other to produce biological effects. Triterpenoid saponins, such as CalundulosideE (Wang et al., 2021d), platycodinD (Lei et al., 2022), and Ginsenoside Rh4 (Wu et al., 2018), and steroidal saponins, like Dioscin (Wang et al., 2014) and Polyphyllin D (Liu et al., 2022b) exert anti-tumor effects by regulating the MAPK signaling pathways (Figure 3F). (1) p38 MAPK: RLTS can activate p38 MAPK and downregulate the protein expressions of CXCR4, MMP2, and MMP9, suppress cell migration, induce apoptosis, and inhibit the proliferation of cancer cells both *in vivo* and *in vitro* (Zhan et al., 2016). Paris Saponin I (PSI) significantly reduces the levels of Bcl-2 and Bcl-xl but promotes those of p-p38 MAPK, Cyto-c, caspase-9, and caspase-3 in lung cancer cells (Liu et al., 2017). (2) ERK1/2: Timosaponin A3 decreased the levels of MMP-9, Bcl-2, Bcl-xl, and VEGF-1 (in pancreatic cancer AsPC-1 cells) by inhibiting the ERK1/2 pathway (Kim et al., 2019). (3) JNK: Protodioscin regulates the key extrinsic apoptotic pathway molecules, including Bcl-2, caspases-8/3/9, -PARP, and Bax (Lin et al., 2018). Protodioscin promotes the expression of cleaved-PARP and cleaved-caspase 3 while decreasing E-cadherin levels (Chen et al., 2022). Moreover, T-17 decreased P62 expression but increased those of P21 and Beclin-1 by regulating the JNK signaling pathway (Xu et al., 2020b).

### APMK signal transduction pathway

AMP-activated protein kinase (AMPK) is a sensor of energy status and regulator of metabolism in eukaryotic cells and comprises three subunits, namely, *α, β*, and *γ*. AMPK-triggered energy imbalance can cause the development of several diseases, including diabetes, inflammation, obesity, and cancer (Paskeh et al., 2022). Among them, AMPK regulates cancer cell metabolism and is a promising anti-cancer target (Samarghandian et al., 2016). Activation of AMPK signaling is associated with an increase in AMP: ATP and ADP: ATP ratios, thus driving the stimulation of AMPK by upstream molecules (LKB1 and CAMKK) (Ashrafizadeh et al., 2021). APMK is activated in the phosphorylated state and its activation is strongly associated with improved survival of patients with breast (Henry et al., 2017), bladder (Tao et al., 2017), and colon (Wei et al., 2016) cancers. Phosphorylated AMPK activates TSC1/2 proteins, thereby inhibiting mTOR kinase activity (Bai et al., 2017). mTOR is a regulator associated with autophagy, and saponins reportedly target autophagy through the AMPK/mTOR signaling pathway to slow the malignant progression of cancer. PGB shows a noticeable pro-autophagy effect on A549 human lung carcinoma cells *in vitro*. Studies have revealed that PGB-activated AMPK phosphorylation inhibits mTOR and AKT activities. Furthermore, PGB increases the levels of lC3-II, Beclin-1, and Bax but decreased those of the mTOR complex (Raptor and Rictor) and Bcl-2 (Yim et al., 2016).

GIT, an important component of *Tribulus longipetalus,* belongs to a family of steroidal saponins. Both *in vivo* and *in vitro* experiments indicate that GIT promotes Bax expression, activities of cleaved Caspase-3 and PARP, and enhances LC3II and P-AMPK expressions in lung cancer cells through the AMPK and AKT signaling pathways, thereby initiating autophagy. Thus, it is a potential apoptosis and autophagy inducer (Liu et al., 2022c). Moreover, DT-13 activates AMPK phosphorylation and reduces the expressions of p-mTOR, p-p70S6K, GLUT1, and p-4EBP1 in the HCT-15 and HT-29 cells (Wei et al., 2019). Tetracyclic triterpenoid AS-IV exerts anti-tumor effects by reducing tumor cell growth, invasion, migration, and angiogenesis. An *in vivo* study showed that AS-IV inhibited the levels of M2 surface marker (CD206) and macrophage markers (PPARγ and Arg-1), MMP9, MMP10, and MMP14. It also significantly downregulated the levels of IL-10 and TGF-β by targeting the AMPK signaling pathway (Xu et al., 2018) (Figure 3G).

### p53 signal transduction pathway

The p53 tumor suppressor is a transcription factor that induces apoptosis. When Afrocyclamin A was added to human prostate cancer cells, the expressions of p53, p21, and Bax were found to increase. The results showed that Afrocyclamin A could effectively induce apoptosis (Sachan et al., 2018). The treatment of HCC cells with dioscin showed that the expressions of P53 and P21 increased while those of CHK2, cyclin B1, and CDK1 were inhibited. Dioscin could effectively block the cell cycle in the G2/M phase through the p53 signaling pathway (Zhang et al., 2018a). These results show that saponins are engaged in the p53 signaling pathway (Figure 3C).

In summary, saponins play essential roles in anti-tumor effects through the regulation of NF-κB, PI3Ks-Akt-mTOR, MAPK, Wnt-β-catenin, JAK-STAT3, APMK, p53, and EGFR signal transduction pathways (Figure 3). The saponins summarized in this review are mainly divided into monomer saponins and total saponin extracts as shown in Tables 1, 2.

**TABLE 1:** Monomer saponins against cerebral ischemia-reperfusion injury. Up arrows indicate upregulation, while the down arrows indicate downregulation.

Comp. and source	Cancer model (s)	Mechanism	Target	Signaling pathway	Refs
Triterpenoid saponins					
Afrocyclamin A, *Androsace umbellata*	Human prostate cancer cell lines (LNCaP, PC-3, and DU145)	Pro-autophagy pro-apoptosis Anti-migration anti-invasion	Bcl-2 ↓ cyclin E/D/B↓ CDK2,CDK4↓	PI3K/Akt/mTOR↓ P53↑	Sachan et al. (2018)
Raddeanin A *Anemone raddeana*	SW480, Caco-2, HT29,LOVOcells, Male nude mice	Pro-apoptosis Anti-proliferation	c-Myc,CyclinD1, p-LRP6↓	Wnt/β-catenin NF-κB↓	Wang et al. (2018b)
	The human osteosarcoma cells (U-2 OS, HOS, MG-63,143B,Saos-2)	Pro-apoptosis Anti-proliferation anti-migration	MMP-2/9,Bcl-2↓ p65↓ caspase-3, Bax↑	NF-κB↓	Ma et al. (2018)
	NSCLC cells of A549	Anti-proliferation pro-apoptosis	caspase-3↑, Bax↑	STAT3↑	Li L et al. (2021)
1C, *Panax ginseng*	Human prostate cancer cell lines (LNCaP, PC3, 22RV1, DU-145, C4-2B, and GES-1 cells)	Pro-apoptosis Anti-proliferation	MDM2, Bcl-2↓ Bax, p53↑ Cleaved caspase-3/9 cleaved PARP↑	Wnt/β-catenin↓	Wang et al. (2018c)
Ginsenoside Rg3, *Panax ginseng*	The pancreatic cancer cell lines (BxPC-3 and AsPC-1) BALB/c nu/nu male mice	Pro-apoptosis	Cleaved caspase-3 Cleaved caspase-9↑ cleaved PARP↑	EGFR/PI3K/AKT↓	Jiang et al. (2017a)
Ziyuglycoside II, *Sanguisorba officinalis* L	HepG2, HuCCT1, BGC-823, HCT116, OE21, PANC-1 cells	Cell cycle arrest Pro-apoptosis oxidative stress	Cleaved caspase-3 cleaved PARP↑	EGFR↓	Zhong et al. (2021)
α-Hederin, *Hedera helix*	The human colon cancer cell line (SW620)	Anti-migration Anti-invasion	Ecapherin,↑ EMT↓	JAK2/STAT3↓	Sun et al. (2018)
Momordin Ic, Fructus Kochiae	HepG2 cell line	Pro-apoptosis Pro-autophagy	Beclin1,LC-3↑	MAPK	Mi et al. (2016)
Hederacolchiside A1, *Pulsatilla chinensis*	Hepatocellular carcinoma cells (Bel-7402, MCF-7)	Pro-apoptosis	Bcl-2↓ Cleaved caspase-3↑	PI3K/Akt/mTOR↓	Wang et al. (2018a)
Jujuboside B, *Zizyphus jujuba*	human colorectal cancer cells (SW1116, SW1463) male Balb/c mice	Pro-apoptosis	Bax/Bcl-2↑, MMP↓, caspase-3 ↑ Cleaved PARP↑ cytochrome C↑	PI3K/Akt↓	Li X et al. (2021)
Saikosaponin-A, *Bupleurum falcatum*	Human cervical cancer HeLa cells Female BALB/c nude mice	Pro-apoptosis	Cleaved caspase-3↑ Bax/Bcl-2↑	PI3K/Akt↓	Du et al. (2021)
Calunduloside E, panax japonicas *Aralia elata* (Miq.) Seem	Human hepatoma cell (HepG2)	Anti-proliferation anti-migration	HMGB1↓,MMPs↓,Cycins,N-cadherin↓ E-cadherin↑	p38/JNK ↑	Wang et al. (2021a)
Platyco-din D, *Pla*-*tycodonis Radix*	human hepatoma cells (SMMC-7721)	Pro-apoptosis	p-AKT,↓ p-JNK↑ p-ERK1/2↓ p-P38 MAPK↑	AKT, ERK1/2↓ JNK,P38MAPK↑	Lei et al. (2022)
Ginsenoside Rh4, *Panax notoginseng*	Human colorectal cancer cells (Caco-2 and HCT116) Nude mice	Pro-apoptosis Pro-autophagy	Cyclin D1↓,CDK4↓ p53,p21,Bax↑ Caspase3,9↑	ROS/JNK/p53↑	Wu et al. (2018)
Astragaloside IV, Radix Astragali	U251 cells Athymic BALB/c mice	Anti-proliferation Anti-migration anti-invasion	Ki67,MMP-2/-9↓ VEGF↓	MAPK/ERK↓	Li et al. (2017)
	Human breast cancer (MDA-MB-231) Female athymic Balb/c nude mice	Anti-invasion	MMP-2/-9↓ VAV3↓	MAPK↓	Jiang et al. (2017b)
	Human monocyte cell line THP-1 Male C57BL/6 J mice	Inhibit metastasis	CD31,VEGFA↓	AMPK	Xu et al. (2018)
**Steroidal saponins**					
Paris Saponin II, *Rhizoma Paridis*	BEAS-2B, NCI-H460,A549	Autophagy pro-apoptosis	cytochrome C,Bax, Cleaved caspase-3/9 LC3-II,Beclin-1↑ Bcl-2↓	PI3K/Akt/mTOR↓	Zhang et al. (2016)
	HT 29, HCT 116 cell lines Female nude mice	pro-apoptosis cell cycle arrest	p65,c-myc, cyclin D1↓	NF-κB↓	Chen et al. (2019)
A-24, *Allium chinense*	SGC-7901 and AGS cells	Autophagy pro-apoptosis	Caspase3,9↑ LC3-II,Beclin-1↑	PI3K/Akt/mTOR↓	Xu et al. (2020a)
Polyphyllin VII, *Paris polyphylla*	Human lung cancer A549 cells	pro-apoptosis	p65↓ caspase-3↑	PI3K/Akt NF-κB↓	He et al. (2020)
DT-13, Dwarf lilyturf	MDA-MB-231,MDA-MB-468 breast cancer cells	Anti-migration	PLOD2 Gp130,OBR↓	JAK/STAT3 PI3K/AKT↓	He et al. (2019a)
	HCT-15, HCT-116, COLO 205, HT-29, SW-620 and SW-480 cells Female BALB/c athymic nude mice	Anti-proliferation	mTOR, P70S6K and 4EBP1↓	AMPK	Wei et al. (2019)
Ophiopogonin B, Ophiopogon japonicus (L.f.) Ker-Gawl	SKOV3, A27800 cells	Anti-proliferation Pro-apoptosis Anti-migration cell cycle arrest	P-STAT3↓	STAT3	Yuan et al. (2022)
Paris Saponin I *Paris polyphylla*	NCI-H1299, NCI-H520, NCI-H460, SCLC NCI-H446	Pro-apoptosis	Cyto-C, Bax Caspase-3/9↑ Bcl-2,Bcl-xl↓	p38 MAPK, ERK, Akt	Liu et al. (2017)
Protodioscin asparagus, yams, the herb fenugreek	Human bladder cancer cell lines 5,637 and T24 Male BALB/c nude mice	Pro-apoptosis Anti-migration anti-invasion	E-cadherin↓ cleaved-PARP cleaved-caspase 3↑	JNK,p38↑	Chen et al. (2022)
	The human cervical cancer cell lines, HeLa and C33A	Pro-apoptosis	caspase-8/3/9, -PARP, Bax↑Bcl-2↓	JNK,p38↑	Lin et al. (2018)
Dioscin, *Polygonatum* plants	The human HEp-2, TU212 and NP69 cell lines	Pro-apoptosis Anti-migration cell cycle arrest anti-invasion	p-53,Bax, Cyto-C Caspase-3/9↑ MMP2/9, cyclina CDK2,Bcl-2↓	MAPK	Si et al. (2016)
	Human hepatocellular carcinoma cell lines (HepG2)	Pro-apoptosis	Bcl-2↓, Bax↑ Caspase-3/9↑	PI3K/Akt/mTOR↓ P53↑	Zhang et al. (2018b)
	Human myeloblast leukemia HL-60 cells	Induced apoptosis	Caspase-3/9↑	p38 MAPK,JNK↑	Wang et al. (2014)
	The murine melanoma B16-F10 Male C57BL/6 mice	Anti-migration, anti-invasion anti-metastasis	IL-4,IL-10↓	JAK2/STAT3↓	Kou et al. (2017)
Timosaponin AIII, *Anemarrhena asphodeloides*	A549/Taxol,A2780/Taxol cells Male BALB/c nude mice	Inhibit cell growth, induced apoptosis	P-gp、PARP,Bcl-2↓ Bax↑	PI3K/Akt/mTOR↓	Song et al. (2019)
Paris saponin I, II, VI, VII, *Rhizoma Paris*	Human umbilical vein endothelial cells (HUVEC)	Anti-angiogenesis	MMP-2↓	PI3K/AKT/mTOR JAK2/STAT3↓	Wang et al. (2018b)
	HepG2,MCF-7, PC-3 cells	Cell cycle arrest Induce apoptosis	MOMP,Bax, cleaved caspase-3/9, Cyto-c↑,CDK1↓	MAPKs PI3K/Akt↓	Long et al. (2015)
Polyphyllin D, *Paris polyphylla* Sm	Human breast cancer cells (MCF-7,MCF10A, MDA-MB-468) BALB/c nude mice	Induced apoptosis	Beclin1, LC3II Cleaved caspase-3↑	JNK1/Bcl-2↑	Liu et al. (2022a)
T-17, *Tupistra chinensis* Baker	SGC-7901, AGS cells	Pro-apoptosis pro-autophagy	Cycline2,P21, Beclin-1,↑ p62↓	JNK↑	Xu et al. (2020b)
Timosaponin A3, *A. as-phodeloides*	Human pancreatic cancer cells (AsPC-1)	Anti-proliferation pro-apoptosis	P21↑,Bcl-2↓, Bcl-XL, CyclinD1 MMP-9,VEGF-1↓	STAT3 ERK1/2↓	Kim et al. (2019)

**TABLE 2:** Total saponin extracts against cancer. Up arrows indicate upregulation, while the down arrows indicate downregulation.

Comp. and source	Cancer model (s)	Mechanism	Target	Signaling pathway	Refs
Triterpenoid saponins					
TBSE, *Bupleurum chinensis* DC.	Human colon cancer cells (SW480, SW620)	Pro-apoptosis	Bcl-2↓ Bax↑ Cleaved caspase-3↑ Cleaved caspase-9↑	PI3K/Akt/mTOR↓	Zhang et al. (2022)
PNS, *Panax notoginseng*	Y79 cells	Pro-apoptosis Anti-proliferation	Bax↑,Bcl-2↓, caspase-3/9 ↑ Cleaved caspase-3↑ Cleaved caspase-9↑	PI3K/Akt↓	Liu et al. (2022b)
CBS, *Conyza blinii* H.Lev	HeLa, MCF-7, HepG-2, MGC-803, A549,BEAS-2B cells female Kunming mice	Pro-apoptosis	Bcl-XL,COX-2, Cyclin D1,MMP-2, MMP-9↓ Cleaved caspase-3 Cleaved caspase-9↑	NF-kB↓	Ma et al. (2017)
PGB *Platycodon grandiflorum*	A549, NCI-H1299	Pro-autophagy	Raptor, Rictor↓ LC3-Ⅱ↑	AMPK	Yim et al. (2016)
RLTS, Radix et Rhizoma Leonticis	SPF mice, HepG2, BEL-7402, A549, SMMC-7721, NCI-H1299, NCI-H460	Anti-proliferation pro-apoptosis	CXCR4, MMP2, M-MP9↓	PI3K/Akt/mTOR↓ p38 MAPK	Zhan et al. (2016)
Total secondary Saponin, *Anemone raddeana* Regel	MCF-7 cells Female BALB/c-nude mice	Anti-proliferation pro-apoptosis	Bax/Bcl-2↑ cytochrome c↑ caspase-3/9↑	PI3K/Akt/mTOR↓	Zhang et al. (2020)
Patrinia villosa Saponins, Herba Patriniae	Human CRC cell (SW480)	Anti-invasion	E-cadherin↓ NF-KBP65↓ N-cadherin↓	NF-kB↓	Xia et al. (2018)
	Human RCC cells (769-P, 786-O)	Inhibit cell growth pro-apoptosis cell cycle arrest	p53↑, p-p38 EGFR, MEK, p38 p-EGFR,p-MEK↓	EGFR/p38	Cai et al. (2017)
Ginsenosides, Korean Red ginseng	528NS cells Mouse	Anti- propagation	LEF1↓, CMYC↓ CCND1↓	Wnt/β-catenin↓	Ham et al. (2019)
**Steroidal saponins**					
SSOJ, *Ophiopogon japonicus* (T.f) Ker-Gawl	Human lung adenocarcinoma A549 cell line	Pro-autophagy	p53,LC3-II/LC3-I ratio,Atg-3, Atg-7 Beclin-1↑ Ki67↓	PI3K/Akt/mTOR↓	Chen et al. (2017)

### Saponins extraction

**FIGURE 4:**
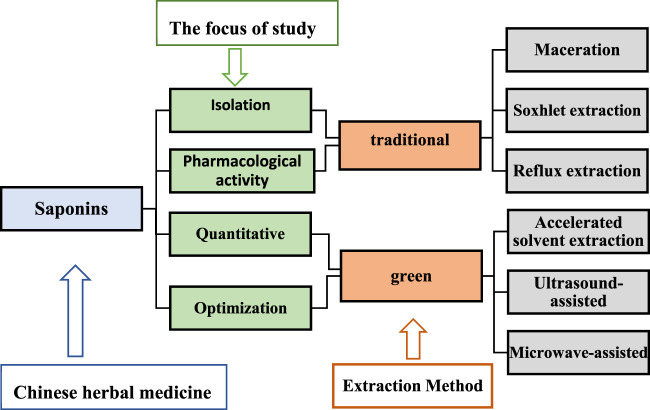
The extraction methods of Chinese materia medica saponins.

Saponins are the main active ingredients of many herbal plants and have a wide range of biological effects. Several extraction methods have been employed to obtain saponins, which are mainly divided into two categories, namely, traditional extraction and green extraction processes (Figure 4). The choice of either method depends mainly on the focus of the study; for saponin isolation and pharmacological activity studies, traditional methods are preferred, while for quantitative and optimization studies, green techniques are employed (Choon et al., 2014). Traditional extraction methods include maceration, soxhlet extraction, and reflux extraction (Ramli et al., 2019). These are dependent on solubility and require high solvent and time depletion (Yusoff et al., 2022), and therefore, have prompted scientific researchers to invent green extraction methods such as ultrasound-assisted, microwave-assisted, and accelerated solvent extraction methods. Ultrasound reportedly increases the solubility of saponins and improves their bioavailability (Navarro Del Hierro et al., 2018b). Ultrasound-assisted extraction has characteristics of less solvent requirement, shorter time, and higher extraction yields, making it a promising alternative method (Fu et al., 2021). With the advancement of scientific research, the exploration of saponin-like components in herbal medicines has increased, the perception of saponins has been redefined to some extent, and their development and application value have been improved.

## Conclusion and perspective

Cancer is a complex pathological process with multifactorial involvement and multiple implicated pathways. The activation or inhibition of many **c**ell signal transduction pathways drives the genesis and development of tumors, and saponins derived from CHM interact with them to inhibit cancer. An ongoing understanding of the role of saponins on signaling pathways involved in cancer development and progression will help us fight cancer in a more precise manner. Therefore, the development of saponins to act against different targets of cancer is of extreme importance. This review summarizes the classification, composition, structure, and extraction methods of saponins. Moreover, the function of saponins in regulating NF-κB, PI3Ks-Akt-mTOR, MAPK, Wnt-β-catenin, JAK-STAT3, APMK, p53, and EGFR signaling pathways are summarized, which may be useful for broadening their anti-cancer activity spectrum. This is expected to enhance researchers’ understanding of the anti-tumor effects of herbal saponins to a certain extent.

Saponins of CHM are a large class of ingredients with low side effects, low cost, easy availability, and significant antitumor activity, and are new antitumor agents worthy of further research and development. This review provides good evidence for its potential application in tumor treatment and the improvement of related diseases. However, existing studies still have some limitations. First, most of the research on Chinese herbal saponins is limited to *in vitro* cell-based or animal experiments, and there is a lack of relevant scientific and standardized clinical experimental studies. The effectiveness and safety of saponins for humans need to be verified. Therefore, in future investigations, clinical studies on the involvement of saponins in cancer prevention and treatment through multiple cell signaling pathways should be conducted to provide a more reliable theoretical basis for their clinical promotion. Second, clinical antitumor drugs are often combined, and in the studies summarized herein, we found that saponins had outstanding anticancer effects when combined with other drugs (Erlotinib/Ginsenoside Rg3, Jiang et al., 2017b), so synergistic effects of between saponins and other components and the mechanisms that regulate the relevant cell signaling pathways warrant further investigation. Saponins are characterized by low bioavailability and corresponding limitations in clinical application, which may be aided by chemical modification and artificial synthesis, as mentioned above (Compound 1C); therefore, in future research, combining modern technical means and existing research results, multidisciplinary crossover in-depth study of the anti-cancer mechanism of saponins, and development of prominent efficacy and clearer action targets of anti-tumor drugs should be explored. The depth and breadth of research on the treatment of cancer by Chinese herbal saponins should be increased in subsequent studies to reflect their role and value in the prevention and treatment of cancer and better promote the development and utilization of these CHM resources.

## Glossary

Akt: Protein kinase B;
AMPK: AMP-activated protein kinase;
AS-IV: Astragaloside IV;
Bcl-xl: B-cell lymphoma-extra-large;
Bax: Bcl-2-associated X;
Bcl-2: B-cell lymphoma-2;
Cyt-c: Cytochrome c;
CDK1: cyclin dependent kinase 1;
Cyclin: cell cycle protein;
CK1α: casein kinase 1α;
CHM: Chinese herbal medicine;
EGFR: Epidermal growth factor receptor;
EMT: epithelial-mesenchymal transition;
ERK: extracellular signal-regulated kinase;
EGFR: epidermal growth factor receptor;
JAK: Janus Kinase;
GSK3β: glycogen synthase kinase 3β;
JNK: c-Jun N-terminal kinase;
LC3: Microtubule-associated protein light chain 3;
MAPK: mitogen-activated protein kinase;
MMP: matrix metalloproteinase;
mTOR: mammalian target of rapamycin;
NF-κB: Nuclear factor kappa B;
NF-κB: nuclear factor kappa-B;
PIP2: phosphatidylinositol bisphosphate;
PIP3: phosphatidylinositol trisphosphate;
p70S6K: Ribosomal protein S6 kinase;
TcF/LEF: T-cell transcription factor/lymphocyte enhancer factor;
PARP: Poly (ADP-ribose) polymerase;
p-Akt: Phosphorylated Protein kinase B;
PI3K: phosphoinositide 3-kinase;
STAT3: signal transducer and activator of transcription-3;
TCM: Traditional Chinese herbal medicine;
Wnt: The wingless-related integration site
